# A novel nano‐immunotherapeutic remodels the immune landscape of malignant pleural effusion: Insights into its mechanism of action through single‐cell RNA‐sequencing

**DOI:** 10.1002/ctm2.774

**Published:** 2022-03-16

**Authors:** Yang Liu, Dawen Zhao

**Affiliations:** ^1^ Department of Biomedical Engineering Wake Forest School of Medicine Winston‐Salem North Carolina USA; ^2^ Department of Cancer Biology Wake Forest School of Medicine Winston‐Salem North Carolina USA

**Keywords:** malignant pleural effusion, nanoparticle immunotherapeutic, single‐cell RNA sequencing, anti‐PD‐L1 immunotherapy

## Abstract

Clinical evidence indicates that the microenvironment in malignant pleural effusion (MPE) is immunologically cold, which impairs tumour immunosurveillance and antitumor immune response to immune checkpoint blockade (ICB). In a recent issue of *Nature Nanotechnology*, Liu et al. demonstrate a new nanotechnological approach to effectively mitigate the immune cold MPE and provide insights into its mechanism of action through single‐cell RNA‐sequencing.

Malignant pleural effusion (MPE) that occurs secondary to metastatic cancer or malignant mesothelioma is the accumulation of fluid in the cavity between the lungs and chest wall. There are over 150 000 new cases of MPE in the United States each year,^1^ and the incidence of MPE is rising worldwide. The development of MPE is indicative of the late stage of cancer and poor prognosis. The accumulation of pleural effusion commonly causes breathlessness, pain, extreme bodyweight loss and reduced physical activity, severely affecting patient's quality of life. The current standard of care for MPE is largely palliative, including catheter drainage of the fluid or chemical/surgical procedures to seal up the cavity.^2^

Recent clinical trials with ICB immunotherapy, e.g., dual anti‐PD‐1 and anti‐CTLA antibodies (Abs), have shown some improvements in overall survival over chemotherapy (18.1 vs 14.1 months in CheckMate 743) in mesothelioma patients.^3^ Clinical cytopathology data indicate that MPE comprises abundant immune cells with tumour‐promoting phenotypes and high levels of immunosuppressive cytokines, which are thus deemed immunologically cold.^4^, ^5^ Mounting evidence suggests that the immune cold tumour microenvironment (TME) impairs the anti‐tumour immune response to ICB. Despite many previous attempts made through intrapleural administration of immunostimulants such as bacterial antigens, pro‐inflammatory cytokines, oncolytic virus or adenoviral cytokine genes,^6^, ^7^, ^8^, ^9^ variable degrees of efficacy have been reported.^4^, ^10^

In the February 2022 issue of *Nature Nanotechnology*, the study by Liu et al. reports a novel nanoparticle immunotherapeutic that is administered directly into the pleural cavity in a mouse MPE model is able to elicit robust antitumor immune responses and synergize with anti‐PD‐L1 Ab against MPE.^11^ The liposomal nanoparticle LNP‐CDN loaded with cyclic dinucleotide (CDN), a potent agonist of stimulators of interferon genes (STING), provides excellent protection of CDN from enzymatic degradation by ENPP1, a phosphodiesterase, which exists at high concentrations in malignant effusions.^12^ Given the prevalent population of tumour associated macrophages and neutrophils in MPE, which contributes markedly to the immune cold MPE, LNP‐CDN is designed to incorporate phosphatidylserine (PS), one type of phospholipid, on the surface of the liposome. Membrane‐exposed PS is known to serve as an ‘eat me’ signal to phagocytes including macrophages, dendritic cells (DCs) and neutrophils.^13^, ^14^, ^15^ Therefore, LNP‐CDN is expected to target CDN to phagocytes and subsequently activate STING signalling and production of type I interferons (IFNs), consequently making the immune cold MPE hot. Moreover, as antigen‐presenting cells (APCs), activated DCs and macrophages can present tumour antigens to prime cytotoxic effector CD8+ T cells.^16^

Unique to MPE, there are two distinct TME compartments co‐existing in the pleural cavity, the effusion and disseminated tumours (carcinomatosis) growing on the cavity surface. Thus, the investigators propose to administer LNP‐CDNs directly into the pleural cavity and pharmacological studies show that intrapleurally administered LNP‐CDNs not only disperse in the fluid but also penetrate well into individual solid tumours, and are subsequently taken up by APCs. Notably, LNP‐CDNs are also detected in APCs in tumour draining lymph nodes (TDLNs), which likely potentiates APC‐mediated cross‐priming of adaptive T‐cell immunity. Therapeutically, intrapleural combination of LNP‐CDNs and anti‐PD‐L1 Abs results in marked reduction of MPE volume and tumour burdens and significantly prolonged survival of the MPE mice. Furthermore, LNP‐CDN is tested in fresh MPE samples obtained from non‐small cell lung cancer (NSCLC) patients, confirming its ability to target human APCs and induce a phenotypic change from M2‐like to M1‐like macrophages, and enhance the tumour cell cytotoxicity of autologous NK and CD8+ T cells in the effusion. Importantly, intrapleural LNP‐CDN alone or in combination with anti‐PD‐L1 Ab is found to be safe without causing immunotoxicity.

To gain insights into the immunological effects of LNP‐CDN, and specifically, how individual immune cell populations respond to LNP‐CDN, the authors conducted scRNA‐seq of MPE samples obtained 48 h after control treatment, anti‐PD‐L1 Ab, LNP‐CDN or a combination of LNP‐CDN and anti‐PD‐L1. As depicted in in t‐stochastic neighbour embedding (t‐SNE) projection (Figure 1), the control MPE contains a large number of myeloid cells including monocytes/macrophages (∼55%) and neutrophils (∼30%), of which M2‐like macrophages (∼82% of the total of macrophages) and N2‐like neutrophils (∼70% of the total of neutrophils) are prevalent subpopulations. Strikingly, intrapleural LNP‐CDN but not anti‐PD‐L1 completely inverts the ratios of M1 to M2 macrophages and N1 to N2 neutrophils, although there is little change in the total number of myeloid cells. LNP‐CDN alone or in combination with anti‐PD‐L1 also induces an increased number of MPE‐infiltrating NK and CD8^+^ T cells (Figure 1) and enhances their tumour cell‐killing activity. In‐depth scRNA‐seq reveals that LNP‐CDN alone or in combination with anti‐PD‐L1 promotes polyfunctional effector NK cells and CD8+T cells and expands the population of stem‐like CD8+T cells. Recent studies have indicated that these CD8+ T subclusters are imperative to sustain durable antitumor response to ICB immunotherapy.^17^, ^18^ Moreover, intrapleural LNP‐CDN is found to upregulate PD‐L1 expression. Together, these unbiased analyses provide mechanistic insights into the immunological and therapeutic effects of LNP‐CDN and support the rational combination with anti‐PD‐L1 ICB.

**FIGURE 1:**
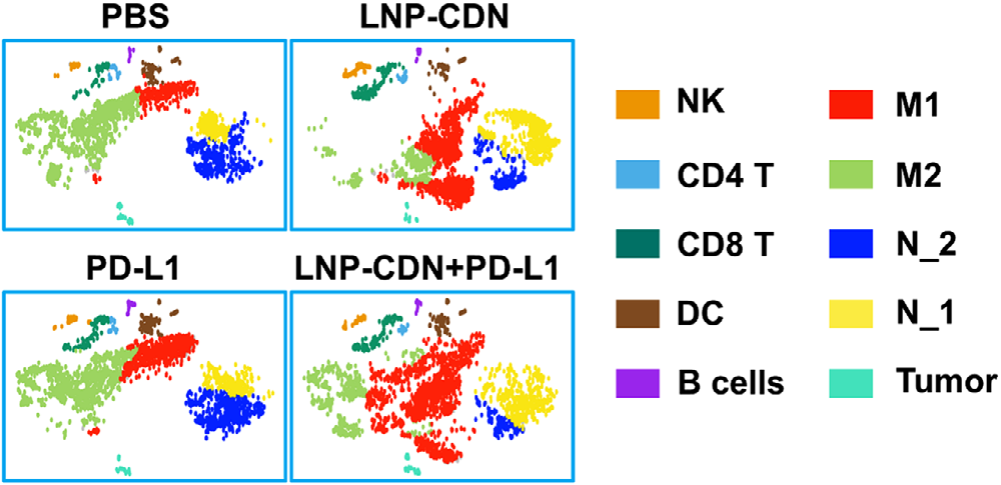
Intrapleural nano‐immunotherapeutic LNP‐CDN makes the immune cold MPE hot. scRNA‐seq of MPE samples reveals that LNP‐CDN induces drastic changes in the immune landscape of MPE.

From a clinical perspective, the nano‐immunotherapeutic developed by Liu and colleagues may have the potential for rapid clinical translation in the form of an immunologic adjuvant. LNP‐CDN can be administered serially by indwelling pleural catheters to remodel the immune landscape favourably for combined ICB immunotherapy such as anti‐PD‐1/PD‐L1 Abs. As the presence of MPE often precludes surgical or chemotherapeutic intervention, successful management of MPE may renew the opportunities for combining with other treatment options to maximize therapeutic efficacy.
